# Carnitine supplementation to obese Zucker rats prevents obesity-induced type II to type I muscle fiber transition and favors an oxidative phenotype of skeletal muscle

**DOI:** 10.1186/1743-7075-10-48

**Published:** 2013-07-10

**Authors:** Aline Couturier, Robert Ringseis, Frank-Christoph Mooren, Karsten Krüger, Erika Most, Klaus Eder

**Affiliations:** 1 Institute of Animal Nutrition and Nutrition Physiology, Justus-Liebig-University Giessen, Heinrich-Buff-Ring 26-32, 35390 Giessen, Germany; 2 Department of Sports Medicine, Justus-Liebig-University Giessen, Kugelberg 62, 35394 Giessen, Germany

**Keywords:** Carnitine, Zucker rat, Muscle fiber transition, Type I fiber, Oxidative capacity, Fatty acid oxidation

## Abstract

**Background:**

In the present study, we tested the hypothesis that carnitine supplementation counteracts obesity-induced muscle fiber transition from type I to type II.

**Methods:**

24 obese Zucker rats were randomly divided into two groups of 12 rats each (obese control, obese carnitine) and 12 lean Zucker rats were selected for lean control group. A control diet was given to both control groups and a carnitine supplemented diet (3 g/kg diet) was given to obese carnitine group for 4 wk. Components of the muscle fiber transformation in skeletal muscle were examined.

**Results:**

The plasma level of carnitine were lower in the obese control group compared to the lean control group and higher in the obese carnitine group than in the other groups (*P* < 0.05). Plasma concentrations of triglycerides and non-esterified fatty acids were increased in obese animals compared to lean animals and the obese carnitine group had lower level compared to the obese control group (*P* < 0.05). The obese carnitine group had an increased number of type I muscle fibers and higher mRNA levels of type I fiber-specific myosin heavy chain, regulators of muscle fiber transition and of genes involved in carnitine uptake, fatty acid transport, β-oxidation, angiogenesis, tricarboxylic acid cycle and thermo genesis in *M. rectus femoris* compared to the other groups (*P* < 0.05).

**Conclusion:**

The results demonstrate that carnitine supplementation to obese Zucker a rat counteracts the obesity-induced muscle fiber transition and restores the muscle oxidative metabolic phenotype. Carnitine supplementation is supposed to be beneficial for the treatment of elevated levels of plasma lipids during obesity or diabetes.

## Background

The different contractile and metabolic functions of individual skeletal muscles in the body are caused by a different muscle fiber distribution. Two major types of muscle fibers, which differ in their myosin heavy-chain (MHC) isoforms and their enzymatic capacity [1], can be distinguished. Type I fibers are mitochondria-rich, have a high oxidative capacity utilizing mostly oxidative phosphorylation [2,3], and are myoglobin-rich with red appearance. Type II fibers (subdivided in IIa, IIb and IId/x) have a lower oxidative capacity due to fewer mitochondria content than type I muscle fibers and thus depend on glycolytic metabolism to generate ATP.

Interestingly, muscle fiber distribution is altered by genetic and/or high fat diet-induced obesity due to induction of fiber transition from type I to type II thereby leading to a change of the muscle’s metabolic phenotype [4-7]. The resulting reduced type I fiber content is associated with mitochondrial dysfunction characterized by an impaired mitochondrial oxidative enzyme capacity of skeletal muscle [8]. Noteworthy, genetic and/or high fat diet-induced obesity and diabetes cause an impairment of carnitine status [9-11], whereas normalization of carnitine status due to carnitine supplementation reverses mitochondrial dysfunction under these conditions [4,9,10,12,13]. At large, these findings indicate that carnitine supplementation through normalizing carnitine status is able to prevent type I to type II fiber transition and thereby the metabolic shift from oxidative to glycolytic of skeletal muscle under unloading conditions or metabolic stress.

Obesity- and diabetes-induced type I to type II fiber transition and decreases of oxidative capacity of skeletal muscle has been explained by down-regulation of peroxisome proliferator-activated receptor γ, coactivator-1α (PGC-1α) and peroxisome proliferator-activated receptor δ (PPARδ) [4,9,14]. Both, PGC-1α and PPARδ are critical regulators of genes involved in type II to type I fiber transition, mitochondrial biogenesis, cellular and mitochondrial fatty acid uptake, β-oxidation, carnitine uptake, tricarboxylic acid cycle, respiratory chain, and angiogenesis [15-20]. Due to these functions PPARδ and PGC-1α are typically higher expressed in oxidative type I muscle fibers than in glycolytic type II muscle fibers [15,21]. Interestingly, carnitine supplementation was reported to increase expression of PGC-1α and PPARδ in rodent models of unloading [4], and genetic and diet-induced obesity and diabetes [10]. Based on these observations we hypothesized that carnitine supplementation through inducing PGC-1α and PPARδ in skeletal muscle counteracts obesity and/or diabetes-induced muscle fiber transition from type I to type II and restores the muscle fiber distribution and the muscle oxidative metabolic phenotype observed during non-obese and non-diabetic states. As a model object we used obese Zucker rats, an established genetic model of obesity, insulin resistance, and metabolic syndrome, which were fed either a carnitine supplemented or a control diet with a low native carnitine concentration for 4 wk. Lean Zucker rats served as healthy non-obese and non-diabetic controls.

## Methods

### Animals and housing

24 male obese (fa/fa) Zucker rats (Crl:ZUC-*Lepr*^*fa*^; Charles River, France) were randomly divided in two groups of 12 rats each. They had an initial body weight of 357 ± 4 (mean ± SEM) g and aged 8–10 weeks. In addition, 12 male heterozygous lean (fa/+) Zucker rats were used for the lean control group. They were also 8–10 weeks old and had an initial body weight of 271 ± 3 (mean ± SEM) g. The rats were kept in Macrolon cages in a controlled environment with a 12-h light–dark cycle. All experimental procedures followed established guidelines for the care and handling of laboratory animals and were approved by the local Animal Care and Use Committee.

### Diets and feeding

The rats received two different semi-purified diets which were composed according to the recommendations of the American Institute of Nutrition (AIN)-93G [22]. The first diet, which contained no carnitine supplement and had a very low carnitine concentration of below < 5 mg carnitine/kg diet, was given to the lean control and the obese control group. The second diet containing 3 g carnitine/kg diet was given to the obese carnitine group. Both diets contained (g/kg diet): corn starch, 530; casein, 200; saccharose, 100; soybean oil, 70; cellulose, 50; minerals, 30; vitamins, 20. Carnitine was added to the obese carnitine diet at the expense of corn starch. The diets were prepared by mixing the dry components and subsequent pelleting using a standard pelleting device (Kahl Laborpressanlage Typ 14–175; Reinbek, Germany). Feed and water was available *ad libitum* and feed intake was recorded every week during the 28 days of the experiment.

### Sample collection

The rats of all groups were decapitated under CO_2_ anesthesia. Blood samples were taken into EDTA polyethylene tubes (Sarstedt, Nürnbrecht, Germany) and plasma was collected by centrifugation (1,100 × g; 10 min, 4°C). *M. rectus femoris* was excised, immediately snap-frozen with liquid nitrogen and stored at −80°C pending analysis.

### Muscle fiber typing

For muscle fiber typing serial cross-sections of 30 μm thickness from *M. rectus femoris* were prepared using a cryostat microtome at −25°C and muscle fibers were identified by their different inactivation of myofibrillar actomyosin ATPase during acid preincubation, using a modified method according to Hämäläinen and Pette [23], as recently described [24].

### Determination of plasma lipids

The concentration of triglycerides in plasma was determined using enzymatic reagent kits (refs. 157609990314 and 113009990314) [25]. Concentration of non-esterified fatty acids (NEFA) in plasma was measured using the NEFA kit from Wako Chemicals (ref. RD291001200R).

### Determination of carnitine levels in plasma and muscle

Tandem mass spectrometry was used for determining the concentrations of free carnitine and acetyl carnitine in plasma and muscle. Total carnitine was calculated as the sum of free carnitine and acetyl-carnitine. Deuterated carnitine-d3 (Cambridge Isotype Laboratories, Andover, MA, USA) was used as internal standard, according the method of Hirche et al. [26].

### RNA isolation and qPCR analysis

Total RNA was isolated from 20 mg skeletal muscle tissue using Trizol™ reagent (Invitrogen, Karlsruhe, Germany) according to the manufacturer’s protocol. Isolated RNA was stored at −80°C. RNA concentration and purity were estimated from the optical density at 260 and 280 nm (Infinite 200 M micro plate reader, Tecan, Männedorf, Switzerland). The integrity of the RNA was also verified by 1% agarose gel electrophoresis, which showed intact bands corresponding to the 18S and 28S ribosomal RNA subunits. cDNA synthesis and qPCR analysis were performed as described recently in detail [27]. Features of gene-specific primer pairs are listed in Table 1. Calculation of gene expression data and normalization by GeNorm normalization factor were carried out as described recently [27]. In this study the three most stable out of six tested potential reference genes were CANX, TOP1 and YWHAZ in *M. rectus femoris* (Table 2). Means and SEM were calculated from normalized expression data for samples of the same treatment group. The mean of the control obese group was set to 1 and means and SEM of the other treatment groups were scaled proportionally. Data on qPCR performance for each gene measured in skeletal muscle are shown in Table 1.

**Table 1:** Characteristics of primers used for qPCR

**Gene symbol**	**Primer sequence (forward, reverse; from 5′ to 3′)**	**NCBI GeneBank**	**Product size (bp)**	**Slope**	**R**^**2#**^	Efficiency*
**(HUGO)**						
ACADL	AAGGATTTATTAAGGGCAAGAAGC	NM_012819	380 bp	−3.88	0.998	1.81
	GGAAGCGGAGGCGGAGTC					
ACADM	CAAGAGAGCCTGGGAACTTG	NM_016986	154 bp	−3.38	0.999	1.98
	CCCCAAAGAATTTGCTTCAA					
ATP5B	GCACCGTCAGAACTATTGCT	NM_134364	203 bp	−3.59	0.999	1.90
	GAATTCAGGAGCCTCAGCAT					
CANX	CCAGATGCAGATCTGAAGAC	NM_172008	175 bp	−2.75	0.999	2.31
	CTGGGTCCTCAATTTCACGT					
CD36	TCGTATGGTGTGCTGGACAT	NM_031561	358 bp	−3.28	0.996	2.02
	GGCCCAGGAGCTTTATTTTC					
CPT1B	GCAAACTGGACCGAGAAGAG	NM_013200	180 bp	−3.32	0.988	2.00
	CCTTGAAGAAGCGACCTTTG					
FABP3	ACCATCCACTGCCGTCTTAC	NM_013177	310 bp	−3.20	0.957	2.05
	CCCCGATGCGTAGGTATTCT					
HK2	GATGGAATCGAGAAGGCCTA,	NM_012735	220 bp	−3.63	1.000	1.89
	GTTTCTTGTAGACGGAGCCA					
LPL	GAGATTTCTCTGTATGGCACA	NM_012598	276 bp	−3.34	0.992	1.99
	CTGCAGATGAGAAACTTTCTC					
MDH1	CAGACAAAGAAGAGGTTGCC,	NM_033235	206 bp	−3.40	0.994	1.97
	CGTCAGGCAGTTTGTATTGG					
MYH1	GCAGACTCTCCCACTGGGCTG	NM_001135158	83 bp	−3.16	0.953	2.07
	GAGCAGCCTCCCCGAAAACGG					
MYH2	GCTGATCGAAATGCTGCTGA	NM_001135157	124 bp	−3.38	0.990	1.98
	GTCAATAGCACTATCCGTGG					
MYH4	CCAGTCCATCCTGATTACTG	NM_019325	74 bp	−3.48	0.988	1.94
	CAAAGTACTGGATGACACGC					
MYH7	ATTGCCGAGTCCCAGGTCAACA	NM_017240	127 bp	−3.24	0.944	2.03
	GCTCCAGGTCTCAGGGCTTCAC					
PFKM	TCCTGGTTGGCTCAATCGAC	NM_031715	297 bp	−3.75	0.998	1.85
	TGTTGAGACGAGAACCACGG					
PKM	ACCTGGGCATTGAGATTCCG	NM_053297	314 bp	−3.69	0.997	1.87
	TCGCGCAAGCTCTTCAAACA					
PPARD	GCAGAGCTATGACCAGGCCTGCA	NM_013141	151 bp	−3.29	0.990	2.01
	GTGCTCTGGTCCCCCGTTGA					
PPARGC1A	CTCTTTGCCCAGATCTTCCT	NM_031347	145 bp	−3.93	0.999	1.80
	ATGTTCGCGGGCTCATTGTT					
PPARGC1B	CATATAAGCCCATGGAGGAG	NM_176075	476 bp	−3.25	0.978	2.03
	CAGCCCAAAGTGCTTTGTGA					
RPL13	CTTAAATTGGCCACGCAGCT	XR_086310	198 bp	−3.48	0.998	1.94
	CTTCTCAACGTCTTGCTCTG					
SDHA	TGGACCTTGTCGTCTTTGG	NM_130428	88 bp	−3.90	0.997	1.80
	TTTGCCTTAATCGGAGGAAC					
SLC2A4	GAGTTATGTGTCCATCGTGG	NM_012751	187 bp	−2.59	0.953	2.40
	CGCAACATACTGGAAACCCA					
SLC22A5	GAACTCACGAGCCTCGCACGC	NM_019269	117 bp	−3.75	0.997	1.85
	TCGTCGTAGTCCCGCATGCC					
SLC25A20	AGCCCACCTGTTATCCACTG	NM_053965	178 bp	−3.32	0.988	2.00
	TGTGCAAAAAGAGCCTTCCT					
SLC27A1	GTATCTGCTGGACCTTCGC	NM_053580	243 bp	−3.48	0.990	1.94
	CATAAATGAGGGCCTTGGCA					
TOP1	GAAGAACGCTATCCAGAAGG	NM_022615	137 bp	−3.33	0.997	2.00
	GCTTTGGGACTCAGCTTCAT					
UCP1	CAGGCTTCCAGTACTATTAGG	NM_012682	181 bp	−3.40	0.983	1.97
	CTCTCCCTGAAGAGAAGTACT					
UCP2	CAAGGAGAGAGTCAAGGGCTA	NM_019354	209 bp	−3.08	0.998	2.11
	GACTCTGAGCCCTTGGTGTAG					
UCP3	CTCGGTACCATCCTGACTAT	NM_013167	149 bp	−3.47	0.982	1.94
	GTTCCTTTGGGGGTGTAGAA					
VEGFA	GTTCATGGACGTCTACCAGC	NM_031836	253 bp	−3.62	0.973	1.89
	GCTATGCTGCAGGAAGCTCA					
VEGFB	GTGTCCCAGTTTGATGGCC	NM_053549	187 bp	−3.45	1.000	1.95
	CGTCAGGACAGCAGCCAC					
YWHAZ	GACGGAAGGTGCTGAGAAA	NM_013011	198 bp	−3.13	0.986	2.09
	GCAGCAACCTCAGCCAAGT					

^#^Coefficient of determination of the standard curve.

*The efficiency is determined by [10^(−1/-slope^].

**Table 2:** Average expression stability ranking of six candidate reference genes

**Ranking**	**Gene**	**M value**
Most stable	YWHAZ	0.056
	TOP1	0.060
	CANX	0.060
	MDH1	0.062
	ATP5B	0.073
Least stable	RPL13	0.074

Ranking of the candidate reference genes according to their stability score M as calculated by the Microsoft Excel-based application GeNorm.

### Western blotting

Homogenates were prepared and protein concentration was determined as described recently [11]. After protein separation by 12.5% SDS-PAGE the proteins were transferred to a nitrocellulose membrane and incubated with primary antibodies against PGC-1α (polyclonal anti-PGC-1α antibody; Millipore, Temecula, CA) and novel organic cation transporter 2 (OCTN2) (polyclonal anti-OCTN2 antibody; Lifespan Bioscience, Inc., Seattle, US) and glyceraldehyde-3-phosphate dehydrogenase (GAPDH) (monoclonal anti-GAPDH antibody, Abcam, Cambridge, UK) as a reference protein. The membranes were washed, and then incubated with a horseradish peroxidase conjugated secondary monoclonal anti-mouse-IgG antibody (Sigma-Aldrich, Steinheim, Germany) for GAPDH and polyclonal anti-rabbit-IgG antibody (DakoCytomation, Glostrup, Denmark) for PGC-1α and OCTN2 at room temperature. Afterwards blots were developed by ECL Select (GE Healthcare, Munich, Germany) and the intensities of the specific bands were detected with a Bio-Imaging system (Syngene, Cambridge, UK) and quantified by Syngene Gene Tools software (nonlinear dynamics).

### Statistics

Statistical analysis of all data was done by one-way ANOVA using the Minitab Statistical Software (Rel. 13.0, State College, PA, USA). Means of the three groups were compared by Fisher’s multiple range tests. Means were considered significantly different for *P* < 0.05. Data presented are shown as means ± SEM.

## Results

### Feed intake and body weight development

Initial and final body weights as well as daily body weight gain were greater in the obese control group and the obese carnitine group than in the lean control group (*P* < 0.05; Table 3). Similarly, feed intake was greater in the obese control group and the obese carnitine group than in the lean control group (*P* < 0.05; Table 3). The feed conversion ratio which describes gram feed per gram body weight gain was increased significantly in the lean control group (*P* < 0.05; Table 3). These parameters did not differ between the two obese groups.

**Table 3:** **Feed intake and body weight gains of lean rats (lean control), obese Zucker rats fed a control diet (obese control) or obese Zucker rats fed a diet supplemented with 3 g/kg diet carnitine (obese carnitine) for 4 wk**^**1**^

	**Lean**	**Obese**	**Obese**
	**control**	**control**	**carnitine**
Feed intake (g/d)	19.7 ± 0.2^c^	25.2 ± 0.5^b^	26.4 ± 0.4^a^
Initial body weight (g)	271 ± 3^b^	357 ± 6^a^	358 ± 7^a^
Final body weight (g)	367 ± 4^b^	501 ± 7^a^	496 ± 9^a^
Daily body weight gain (g)	3.35 ± 0.08^b^	5.03 ± 0.13^a^	4.85 ± 0.14^a^
Feed conversion ratio (g feed/g body weight)	5.90 ± 0.11^a^	5.02 ± 0.09^b^	5.18 ± 0.18^b^

^1^Data are expressed as means ± SEM, n = 12 rats/group. Means in a row without a common letter differ (*P* < 0.05).

### Concentration of carnitine in plasma and M. rectus femoris

In line with recent observations, the obese control group had lower concentrations of free carnitine, acetyl-carnitine and total carnitine (= sum of free carnitine and acetyl-carnitine) in plasma and rectus femoris muscle than the lean control group (*P* < 0.05; Table 4**)**. Due to carnitine supplementation concentrations of free carnitine, acetyl-carnitine and total carnitine in plasma and rectus femoris muscle were greater in the obese carnitine group than in the lean control group (*P <* 0.05; Table 4).

**Table 4:** **Plasma and muscle (*M. rectus femoris*) concentrations of carnitine, plasma concentrations of TG and NEFA and liver concentration of TG in lean rats (lean control), obese Zucker rats fed a control diet (obese control) or obese Zucker rats fed a diet supplemented with 3 g/kg diet carnitine (obese carnitine) for 4 wk**^**1**^

	**Lean**	**Obese**	**Obese**
	**control**	**control**	**carnitine**
*Plasma (μmol/l)*			
Total carnitine	62.3 ± 1.9^b^	40.0 ± 1.1^c^	90.5 ± 2.9^a^
Free carnitine	50.8 ± 1.6^b^	33.7 ± 1.2^c^	73.2 ± 2.5^a^
Acetyl-carnitine	11.5 ± 0.9^b^	6.3 ± 0.3^c^	17.3 ± 0.8^a^
*M. rectus femoris (nmol/g)*			
Total carnitine	919 ± 13^b^	752 ± 13^c^	1165 ± 19^a^
Free carnitine	742 ± 12^b^	590 ± 9^c^	937 ± 21^a^
Acetyl-carnitine	176 ± 4^b^	161 ± 4^b^	228 ± 6^a^
*Plasma (mmol/l)*			
TG	1.42 ± 0.06^c^	6.35 ± 0.18^a^	4.42 ± 0.26^b^
NEFA	0.73 ± 0.06^c^	3.53 ± 0.16^a^	2.41 ± 0.18^b^
*Liver (μmol/g)*			
TG	10.2 ± 0.9^b^	87.7 ± 13.7^a^	65.6 ± 8.1^a^

^1^Data are expressed as means ± SEM, n = 12 rats/group. Means in a row without a common letter differ (*P* < 0.05).

### Lipid concentrations in plasma and liver

As expected, rats of the obese groups had greater levels of TG and NEFA in plasma and TG in the liver than those of the lean group (*P* < 0.05; Table 4). Due to carnitine supplementation, however, concentrations of TG and NEFA in plasma and TG in the liver were approximately 25–30% lower in the obese carnitine group than in the obese control group (*P* < 0.05; Table 4).

### Fiber type composition and expression of myosin heavy chain isoforms in M. rectus femoris

**Figure 1:**
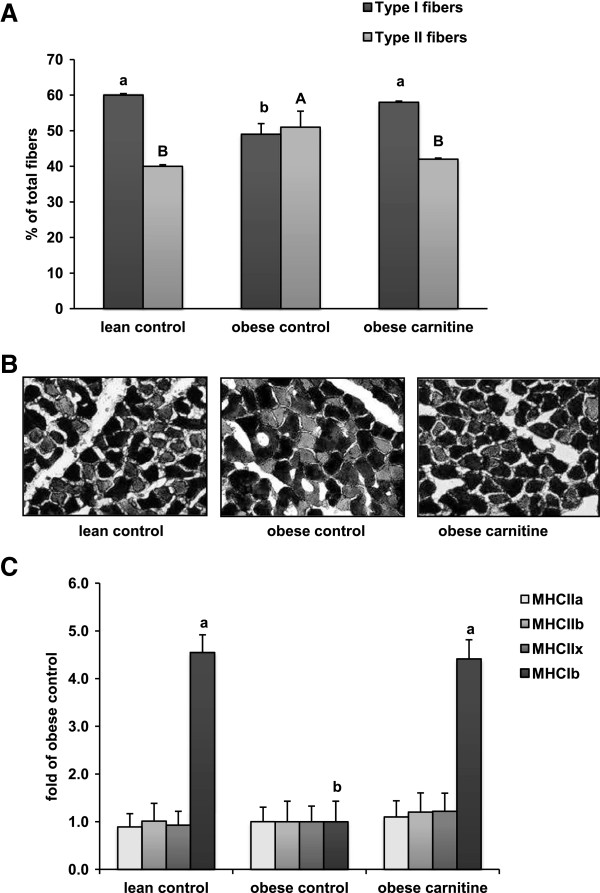
**Fiber distribution of *M. rectus femoris *of lean rats (lean control), Zucker rats fed a control diet (obese control) or Zucker rats fed a diet supplemented with 3 g/kg diet carnitine (obese carnitine) for 4 wk. (A)** muscle fiber type composition, **(B)** fiber type-specific cross-sectional area, **(C)** relative mRNA expression of myosine heavy chain isoforms. Bars represent means ± SEM, n = 12 rats/group. Means without a common letter differ (*P* < 0.05).

Muscle fiber typing revealed an approximately 18% lower percentage of type I muscle fibers in rectus femoris muscle in the obese control group than in the lean control group. Interestingly, the type I muscle fiber percentage in rectus femoris muscle did not differ between the obese carnitine group and the lean control group indicating that carnitine supplementation prevented obesity-induced type I to type II fiber transition (*P* < 0.05; Figure 1A, B). In contrast, the type II fiber percentage in rectus femoris muscle was greater in the obese control group than in the other two groups (*P* < 0.05; Figure 1A, B), but it did not differ between the obese control group and the lean control group. The composition of muscle fiber types in rectus femoris muscle did not differ between the lean control and the obese carnitine group. Relative mRNA levels of genes encoding the different myosin heavy-chain II isoforms (MHCIIa encoded by MYH2, MHCIIb encoded by MYH4, MHCIIx encoded by MYH1) did not differ between the groups (Figure 1C). However, the relative mRNA level of MHCIb (encoded by MYH7), which encodes the type I fiber specific myosin heavy chain isoform; in rectus femoris muscle was greater in the lean control and the obese carnitine group than the obese control group (*P* < 0.05; Figure 1B).

### Expression of genes involved in muscle fiber transformation in M. rectus femoris

The mRNA levels of the main regulators of muscle fiber transformation PPARδ (encoded by PPARD), PGC-1α (encoded by PPARGC1A) and PGC-1β (encoded by PPARGC1B) in rectus femoris muscle were greater in the obese carnitine than in obese control group (*P* < 0.05; Table 5). The lean control and the obese control group did not differ with regard to these genes.

**Table 5:** **Relative mRNA levels of genes involved in carnitine uptake, fatty acid transport, fatty acid utilization, and glucose uptake and glycolysis in *M. rectus femoris *of lean rats (lean control), obese Zucker rats fed a control diet (obese control) or obese Zucker rats fed a diet supplemented with 3 g/kg diet carnitine (obese carnitine) for 4 wk**^**1**^

	**Lean**	**Obese**	**Obese**
	**control**	**control**	**carnitine**
	Fold of obese control		
*Muscle fiber transformation*			
PPARD	1.11 ± 0.19^b^	1.00 ± 0.12^b^	4.18 ± 0.58^a^
PPARGC1A	1.21 ± 0.17^b^	1.00 ± 0.16^b^	2.14 ± 0.42^a^
PPARGC1B	0.78 ± 0.06^b^	1.00 ± 0.24^b^	2.02 ± 0.24^a^
*Carnitine uptake*			
SLC22A5	0.92 ± 0.12^b^	1.00 ± 0.08^b^	1.90 ± 0.25^a^
*Fatty acid transport and uptake*			
FABP3	1.05 ± 0.28^b^	1.00 ± 0.34^b^	2.18 ± 0.46^a^
SLC27A1	1.19 ± 0.34	1.00 ± 0.23	1.46 ± 0.30
CD36	0.71 ± 0.14^b^	1.00 ± 0.11^b^	1.52 ± 0.20^a^
LPL	1.14 ± 0.22^b^	1.00 ± 0.28^b^	2.22 ± 0.35^a^
*β-oxidation*			
ACADM	1.79 ± 0.44^b^	1.00 ± 0.20^b^	3.39 ± 0.86^a^
ACADL	1.11 ± 0.23^b^	1.00 ± 0.24^b^	2.25 ± 0.61^a^
*Carnitine shuttle*			
CPT1B	1.25 ± 0.31	1.00 ± 0.35	1.49 ± 0.27
SLC25A20	1.46 ± 0.14^a^	1.00 ± 0.07^b^	1.39 ± 0.13^a^
*Glucose uptake and glycolysis*			
SLC2A4	1.41 ± 0.34	1.00 ± 0.32	1.52 ± 0.18
HK2	1.32 ± 0.37^b^	1.00 ± 0.51^b^	3.11 ± 0.18^a^
PKM	1.87 ± 0.94	1.00 ± 0.33	2.36 ± 0.46
PFKM	1.20 ± 0.51	1.00 ± 0.32	1.23 ± 0.25

^1^Data are expressed as means ± SEM, n = 12 rats/group. Means and SEM of the other groups were presented as fold of the obese control group, which mean was set to 1. Means in a row without a common letter differ (*P* < 0.05).

**Figure 2:**
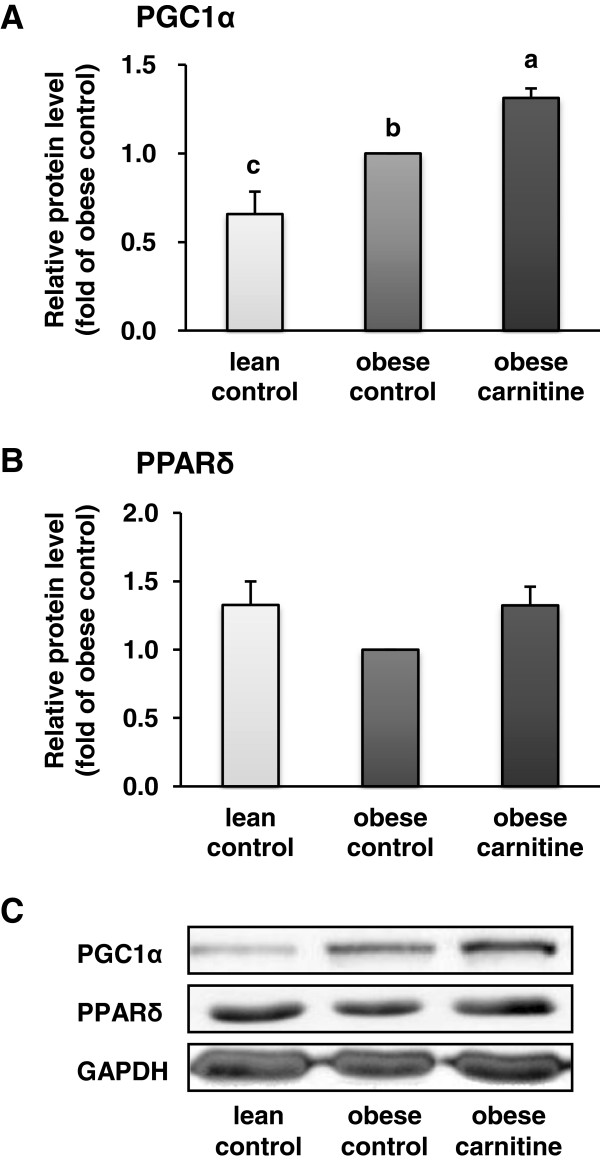
**Relative protein level of PGC-1α (A) and PPARδ (B) in *M. rectus femoris *of lean rats (lean control), Zucker rats fed a control diet (obese control) or Zucker rats fed a diet supplemented with 3 g/kg diet carnitine (obese carnitine) for 4 wk.** Bars represent means ± SEM, n = 6/group. Means without a common letter differ (*P* < 0.05). **(C)** Representative immunoblots specific to PGC-1α, PPARδ and GAPDH as internal control are shown for one animal per group; immunoblots for the other animals revealed similar results.

The relative protein level of PGC-1α in rectus femoris muscle was also greater in the obese carnitine group than in the obese control and the lean control group (*P* < 0.05; Figure 2). The relative protein level of PPARδ in rectus femoris muscle was 33% higher in the obese carnitine group than in the obese control group but this effect was not significant (Figure 2).

### Expression of genes involved in carnitine uptake, fatty acid transport, fatty acid utilization, and glycolysis in M. rectus femoris

The mRNA levels of genes involved in carnitine uptake [SLC22A5 encoding organic cation/carnitine transporter (OCTN2)], fatty acid transport and uptake (FABP3 encoding fatty acid binding protein 3, CD36 encoding fatty acid translocase/CD36, LPL encoding lipoprotein lipase), β-oxidation (ACADL, ACADM encoding long chain acyl-CoA dehydrogenase and medium chain acyl-CoA dehydrogenase, respectively), carnitine shuttle (SLC25A20 encoding carnitine/acylcarnitine translocase), and glycolysis (HK2 encoding hexokinase 2) in rectus femoris muscle were greater in the obese carnitine group than in the obese control group (*P* < 0.05; Table 5). The mRNA levels of SLC27A1 encoding fatty acid transport protein, CPT1B (encoding carnitine-palmitoyl transferase 1b), SLC2A4 encoding glucose transporter-4, PKM (pyruvate kinase, muscle), and PFKM (encoding phosphofructokinase, muscle) in rectus femoris muscle were numerically greater in the obese carnitine group than in the obese control group, but the differences were not significant (Table 5).

**Figure 3:**
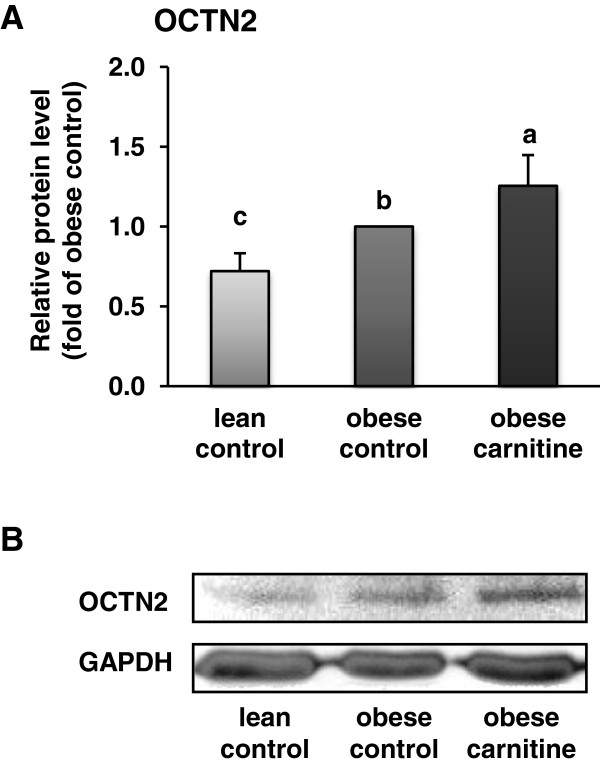
**Relative protein level of OCTN2 in *M. rectus femoris *of lean rats (lean control), Zucker rats fed a control diet (obese control) or Zucker rats fed a diet supplemented with 3 g/kg diet carnitine (obese carnitine) for 4 wk. (A)** Bars represent means ± SEM, n = 6/group. Means without a common letter differ (*P* < 0.05). **(B)** Representative immunoblots specific to OCTN2 and GAPDH as internal control are shown for one animal per group; immunoblots for the other animals revealed similar results.

The relative protein level of OCTN2 in rectus femoris muscle was also greater in the obese carnitine group than in the other groups (*P* < 0.05; Figure 3).

### Expression of genes involved in angiogenesis, tricarboxylic acid cycle and mitochondrial respiratory chain and thermo genesis in M. rectus femoris

Relative mRNA levels of genes encoding vascular endothelial growth factor b (VEGFB), succinate dehydrogenase subunit A (SDHA) and uncoupling proteins (UCP1 and UCP2) in rectus femoris muscle were greater in the obese carnitine group than in the obese control and the lean control group (*P* < 0.05; Table 6). The mRNA level of UCP3 in rectus femoris muscle was numerically greater in the obese carnitine group than in the obese control and lean control group, but this effect was not significant (Table 6).

**Table 6:** **Relative mRNA levels of genes involved in angiogenesis, mitochondrial respiratory chain and uncoupling proteins in *M. rectus femoris *of lean rats (lean control), obese Zucker rats fed a control diet (obese control) or obese Zucker rats fed a diet supplemented with 3 g/kg diet carnitine (obese carnitine) for 4 wk**^**1**^

	**Lean**	**Obese**	**Obese**
	**control**	**control**	**carnitine**
	Fold of obese control		
*Angiogenesis*			
VEGFA	0.81 ± 0.18	1.00 ± 0.30	1.30 ± 0.37
VEGFB	1.62 ± 0.35^ab^	1.00 ± 0.19^b^	2.21 ± 0.28^a^
*TCA cycle and respiratory chain*			
SDHA	1.26 ± 0,25^ab^	1.00 ± 0.24^b^	2.18 ± 0.40^a^
*Thermogenesis*			
UCP1	1.67 ± 0.35^b^	1.00 ± 0.12^b^	7.06 ± 1.27^a^
UCP2	1.07 ± 0.23^b^	1.00 ± 0.24^b^	3.39 ± 0.34^a^
UCP3	0.97 ± 0.20	1.00 ± 0.25	1.92 ± 0.70

^1^Data are expressed as means ± SEM, n = 12 rats/group. Means and SEM of the other groups were presented as fold of the obese control group, which mean was set to 1. Means in a row without a common letter differ (*P* < 0.05).

## Discussion

In the present study we tested the hypothesis that carnitine supplementation to obese Zucker rats counteracts the obesity-induced muscle fiber transition from type I to type II and, thereby, improves fatty acid utilization in skeletal muscle. The dietary carnitine dosage (3 g/kg diet) fed to the rats related to 156 to 216 mg/kg body weight based on an average daily feed consumption of 26 g and a body weight of 360 (initial) to 500 (final) g. This carnitine dosage is slightly higher when compared to that used in clinical studies with human subjects with different metabolic disorders in which carnitine dosages of up to 4 g/d corresponding to 60 mg/kg body weight for an individual weighing 70 kg were found to be effective [28]. A key finding of the present study is that carnitine supplementation to obese rats resulted in an increased number of type I fibers and a decreased number of type II fibers in rectus femoris muscle when compared to non-supplemented obese rats. This indicates that carnitine induces type II to type I fiber transition in femoris muscle of obese rats which was also confirmed by the finding that the type I fiber specific MYH7 mRNA level in rectus femoris muscle was markedly elevated in the obese carnitine group. Interestingly, the fiber type distribution of rectus femoris muscle was similar between the obese carnitine and the lean control group, whereas rectus femoris muscle of the obese control group had a lower number of type I fibers and a greater number of type II fibers compared to that of the lean control group. A reduction of type I fibers and a lower oxidative enzyme activity in muscle of obese and diabetic rodent models compared to lean models has been well documented [6,7]. In addition, several studies reported that obese subjects have a decreased proportion of type I muscle fibers and an overall decrease in mitochondrial enzymes indicating that muscle oxidative capacity is impaired in obese subjects [29-32], which likely contributes to the impaired whole body fatty acid utilization and the elevated blood lipid levels in these subjects. Moreover, it was shown that insulin sensitivity correlates positively with the proportion of type I muscle fibers and negatively with the proportion of type II muscle fibers [29-31]. There is a large body of evidence that carnitine supplementation improves glucose tolerance in insulin resistant and/or diabetic humans (reviewed by [28]). The observed up-regulation of genes in involved in glycolysis (HK2) by carnitine in muscle of the obese rats is also supportive of a beneficial effect of carnitine on glucose homeostasis. Insulin resistant and/or diabetic subjects are likely particularly sensitive to carnitine supplementation because diabetic subjects were reported to have diminished plasma free carnitine concentrations, even though the levels were still within or only slightly below the physiological range (25–50 μmol/l) reported for healthy subjects [33-35]. Given the observed increase in type I muscle fiber proportion in obese rats and the relationship between type I muscle fiber proportion and insulin sensitivity it is not unlikely that the improvement of glucose tolerance and insulin sensitivity by carnitine supplementation in obese and diabetic subjects is due to the effect of carnitine on muscle fiber distribution. Even though a very recent study reported that carnitine supplementation at a non-physiologically high dosage promoted intestinal formation of the proatherogenic trimethylamine-N-oxide and accelerated atherosclerosis development in mice [36], the fact that none of the carnitine supplementation studies in humans reported any adverse effects even at very high dosages (e.g., 4 g oral carnitine) indicates that safety concerns with carnitine supplementation are unfounded. Collectively, the present findings corroborate our hypothesis that carnitine supplementation to obese rats counteracts the obesity-induced muscle fiber transition from type I to type II and is able to restore the muscle fiber distribution and the oxidative metabolic phenotype observed in lean animals.

This study showed that carnitine supplementation in obese rodents resulted in a higher type I muscle fiber content compared to obese rats without carnitine supplementation. The observed decrease of carnitine levels in plasma and muscle in the obese control group is consistent with observations from recent studies showing that whole body carnitine status is strongly compromised in rodent models of genetic and diet-induced obesity and diabetes [10]. As the main reason for this phenomenon an impaired hepatic carnitine biosynthesis has been identified [10,11]. In contrast, carnitine supplementation to obese and/or diabetic rodent models is able to restore the carnitine status to normal [10] or even supraphysiological levels as shown herein. Although our observations do not proof a causal link between carnitine status and muscle fiber distribution, our results suggest that the carnitine-induced change in muscle fiber distribution is due to an improvement of carnitine status.

In addition, the present study shows that the carnitine-induced change in the contractile phenotype of skeletal muscle of obese rats is also accompanied by a change in the metabolic phenotype. In agreement with the high content of mitochondria and the preferential use of fatty acids for energy production of type I fibers, genes involved in fatty acid transport and uptake (FABP3, CD36, LPL), β-oxidation (ACADL, ACADM), carnitine shuttle (SLC25A20), carnitine uptake (SLC22A5) and TCA cycle and respiratory chain (SDHA) in femoris muscle were strongly up-regulated in the obese carnitine compared to the obese control group. In line with our findings in skeletal muscle, carnitine supplementation was also shown to stimulate TCA activity, mitochondrial respiration and ATP production in the diabetic rat heart [37,38]. Thus, these carnitine-induced effects on gene expression of rectus femoris muscle are indicative of an improved capacity of the muscle for oxidative utilization of fatty acids. Since skeletal muscle significantly contributes to whole-body fatty acid utilization, the improved oxidative metabolic phenotype of skeletal muscle at least partially explains the pronounced NEFA- and TG-lowering effects of carnitine supplementation observed in the present rat model of obesity and insulin resistance but also in other studies [39].

To gain insight into the mechanisms underlying muscle fiber transition we determined the mRNA and/or protein levels of the main regulators of fiber composition. The two main regulators of type II to type I fiber transition, mitochondrial biogenesis, and oxidative enzyme expression, PPARδ and PGC-1α, but also PGC-1β, which induces similar effects as PGC-1α on the contractile and metabolic phenotype of skeletal muscle [40,41], were clearly stronger expressed in rectus femoris muscle of the obese carnitine group than in the obese control group. Regarding that PGC-1α and PGC-1β also regulate angiogenesis through inducing the expression of angiogenic factors like VEGFs, we also determined transcript levels of VEGFA (encoding VEGFa) and VEGFB (encoding VEGFb). VEGFb particularly favors the utilization of fatty acids not only by increasing capillary density and thereby blood perfusion but also by inducing the expression of fatty acid transport proteins (FATPs, CD36) [42]. Correspondingly, VEGFb is mainly expressed in tissues with high mitochondria content and with preferential use of fatty acids as energy source like oxidative skeletal muscle, heart, and brown adipose tissue [43]. The observation that the relative mRNA level of VEGFB was markedly elevated in the obese carnitine group is therefore in line with the induction of PGC-1α and PGC-1β and provides a further indication for the improved oxidative phenotype of rectus femoris muscle in rats of the obese carnitine group. Collectively, our findings strongly suggest that the up-regulation of genes encoding PGC-1α, PGC-1β, and PPARδ in rectus femoris muscle by L-carnitine supplementation is responsible for the observed type II to type I fiber transition and the oxidative metabolic phenotype of skeletal muscle of obese Zucker rats.

In conclusion, the results of this study demonstrate that carnitine supplementation to obese Zucker rats significantly improves carnitine status, counteracts the obesity-induced muscle fiber transition from type I to type II and favors an oxidative metabolic phenotype of skeletal muscle which preferentially uses fatty acids as energy source. The enhanced capacity of skeletal muscle to utilize fatty acids was demonstrated by the carnitine-induced up-regulation of genes involved in fatty acid uptake and transport, carnitine uptake, fatty acid β-oxidation, mitochondrial fatty acid uptake (carnitine shuttle system) and tricarboxylic acid cycle. These metabolic changes in skeletal muscle are likely to contribute to the pronounced NEFA- and TG-lowering effects of carnitine supplementation in obese Zucker rats. Therefore, carnitine supplementation is supposed to be beneficial for the treatment of elevated levels of metabolic fuels (e.g., fatty acids) which are frequently found in subjects with obesity, insulin resistance, diabetes or metabolic syndrome.

## Competing interests

The authors declare that they have no competing interests.

## Authors’ contributions

AC, RR and KE designed research and coordinated the study; AC carried out the molecular biological analyses; EM carried out the carnitine analyses; AC, KK and FCM performed muscle fiber typing. AC and KE wrote the paper. KE had primary responsibility for final content. All authors read and approved the final manuscript.
