# Correction: Carnitine supplementation to obese Zucker rats prevents obesity-induced type I to type II muscle fiber transition and favors an oxidative phenotype of skeletal muscle

**DOI:** 10.1186/1743-7075-11-16

**Published:** 2014-04-15

**Authors:** Aline Couturier, Robert Ringseis, Frank-Christoph Mooren, Karsten Krüger, Erika Most, Klaus Eder

**Affiliations:** 1 Institute of Animal Nutrition and Nutrition Physiology, Justus-Liebig-University Giessen, Heinrich-Buff-Ring 26-32, 35390 Giessen, Germany; 2 Department of Sports Medicine, Justus-Liebig-University Giessen, Kugelberg 62, 35394 Giessen, Germany

## Correction

Due to an error, this article [1] was published with an incorrect title. The correct title of the article is ‘Carnitine supplementation to obese Zucker rats prevents obesity-induced type I to type II muscle fiber transition and favors an oxidative phenotype of skeletal muscle’. Readers should be aware that this article may still be indexed under its previous, incorrect title ‘Carnitine supplementation to obese Zucker rats prevents obesity-induced type II to type I muscle fiber transition and favors an oxidative phenotype of skeletal muscle’.
